# Molecular Pathology Analysis of SARS-CoV-2 in Syncytiotrophoblast and Hofbauer Cells in Placenta from a Pregnant Woman and Fetus with COVID-19

**DOI:** 10.3390/pathogens10040479

**Published:** 2021-04-15

**Authors:** Denise Morotti, Massimiliano Cadamuro, Elena Rigoli, Aurelio Sonzogni, Andrea Gianatti, Cristina Parolin, Luisa Patanè, David A. Schwartz

**Affiliations:** 1 Department of Pathology, Papa Giovanni XXIII Hospital, 24127 Bergamo, Italy; dmorotti@asst-pg23.it (D.M.); erigoli@asst-pg23.it (E.R.); asonzogni@asst-pg23.it (A.S.); agianatti@asst-pg23.it (A.G.); 2 Medical Genetics Laboratory, Papa Giovanni XXIII Hospital, 24127 Bergamo, Italy; 3 Department of Molecular Medicine, University of Padova, 35122 Padova, Italy; massimiliano.cadamuro@gmail.com (M.C.); cristina.parolin@unipd.it (C.P.); 4 Obstetrics and Gynecology Department, Papa Giovanni XXIII Hospital, 24127 Bergamo, Italy; lpatane@asst-pg23.it; 5 Department of Pathology, Medical College of Georgia, Augusta University, Augusta, GA 30912, USA

**Keywords:** Hofbauer cell, SARS-CoV-2, placenta, COVID-19, fetal infection, coronavirus, transplacental transmission, intervillositis, coronavirus, villous macrophages, maternal-fetal transmission, coronavirus disease 2019, ACE2, TMPRSS2, severe acute respiratory syndrome coronavirus 2

## Abstract

A small number of neonates delivered to women with SARS-CoV-2 infection have been found to become infected through intrauterine transplacental transmission. These cases are associated with a group of unusual placental pathology abnormalities that include chronic histiocytic intervillositis, syncytiotrophoblast necrosis, and positivity of the syncytiotrophoblast for SARS-CoV-2 antigen or RNA. Hofbauer cells constitute a heterogeneous group of immunologically active macrophages that have been involved in transplacental infections that include such viral agents as Zika virus and human immunodeficiency virus. The role of Hofbauer cells in placental infection with SARS-CoV-2 and maternal-fetal transmission is unknown. This study uses molecular pathology techniques to evaluate the placenta from a neonate infected with SARS-CoV-2 via the transplacental route to determine whether Hofbauer cells have evidence of infection. We found that the placenta had chronic histiocytic intervillositis and syncytiotrophoblast necrosis, with the syncytiotrophoblast demonstrating intense positive staining for SARS-CoV-2. Immunohistochemistry using the macrophage marker CD163, SARS-CoV-2 nucleocapsid protein, and double staining for SARS-CoV-2 with RNAscope and anti-CD163 antibody, revealed that no demonstrable virus could be identified within Hofbauer cells, despite these cells closely approaching the basement membrane zone of the infected trophoblast. Unlike some other viruses, there was no evidence from this transmitting placenta for infection of Hofbauer cells with SARS-CoV-2.

## 1. Introduction

At the beginning of the coronavirus disease 2019 (COVID-19) pandemic, pregnant women in China were reported to develop an infection with the etiological agent—a novel coronavirus termed severe acute respiratory syndrome coronavirus 2 (SARS-CoV-2) [1]. Because of this, there was great anxiety about whether SARS-CoV-2 could be vertically transmitted to the fetus prior to delivery [2,3]. The identification of increasing numbers of pregnant mothers with COVID-19 giving birth to neonates who were also found to test positive for SARS-CoV-2 [3,4,5,6] raised the question of how and when these infants had acquired their infection, including whether they had become infected through vertical transmission, and whether the virus could be transmitted prior to delivery through the placenta [7,8,9].

It has recently been demonstrated that intrauterine transplacental transmission of SARS-CoV-2 to the fetus can occur in infected pregnant women [10,11,12,13,14,15]. In those cases where both pregnant mothers and their neonates test positive for COVID-19, Schwartz et al. [16] have recommended criteria for diagnosing transplacental transmission of maternal-fetal of SARS-CoV-2. These are based upon the pathological demonstration of the virus in fetal-derived tissues of the placenta, including syncytiotrophoblast, chorionic villus stromal and endothelial cells, using immunohistochemistry or in situ RNA hybridization methods [16,17]. Utilizing these criteria, there have been multiple neonates, both stillborn and liveborn, that appear to have acquired transplacental COVID-19 infection prior to delivery. An examination of placentas from fetuses having acquired SARS-CoV-2 infection via transplacental transmission has revealed a strikingly similar pattern of unusual pathological findings among these cases that include chronic histiocytic intervillositis and syncytiotrophoblast necrosis [18,19].

Hofbauer cells are a morphologically and antigenically heterogeneous group of macrophages that reside in the stroma of the chorionic villi throughout pregnancy. These villous macrophages have been implicated in the pathogenesis of several pathogens (termed TORCH agents) capable of intrauterine transplacental transmission, including Zika virus, human immunodeficiency virus (HIV), *Trypanosoma cruzi*, and others [20]. In order to better understand the potential mechanisms of SARS-CoV-2 transplacental transmission, we utilized molecular pathology techniques to evaluate viral distribution within chorionic villi of a placenta having extensive syncytiotrophoblast infection from a maternal-fetal dyad testing positive for the virus, with particular attention to Hofbauer cell involvement, and discuss the findings in terms of mechanisms of intrauterine transmission.

## 2. Materials and Methods

### 2.1. Ethical Statement

The patient signed an informed consent form approving the use of tissue for the present study and publication of the data, and Institutional Review Board approval was obtained according to the guidelines of the Papa Giovanni XXIII Hospital in Bergamo, Italy.

### 2.2. Patient History

A pregnant woman, at 37 weeks and 6 days gestation, presented to the hospital with cough, fever, and had a positive nasopharyngeal (NP) swab for SARS-CoV-2 that was diagnosed using RT-PCR—details of the obstetrical history and delivery have been previously reported [11]. The mother wore a mask during the labor, delivering a 2660 g neonate by spontaneous vaginal delivery. The newborn had an umbilical cord pH of 7.28 and Apgar scores of 9 at 1 min and 10 at 5 min. Skin-to-skin contact was not permitted between the mother and her newborn. Immediately following delivery, the neonate had a positive test using RT-PCR for SARS-CoV-2 from an NP swab. Repeat testing for SARS-CoV-2 was also positive at 24 h and at 7 days of life. The placenta was sent to the Pathology Department for evaluation and diagnosis. The infant was asymptomatic, had an uneventful hospital course, and was discharged home on the 10th day of life.

### 2.3. Hematoxylin and Eosin Staining

Hematoxylin and eosin (H&E) staining of paraffin-embedded tissue sections (4 µm) was performed with the DAKO CoverStainer (DAKO, Glostrup, Denmark), an automated slide processing system. The instrument was combined with DAKO Ready-to-Use (RTU) reagents, and DAKO validated pre-optimized protocols (DAKO, Glostrup, Denmark). Images were captured using Axio Zeiss Scope A1 microscope (Zeiss, Oberkochen, Germany).

### 2.4. Immunohistochemical Staining

Four micrometer-thick formalin-fixed paraffin-embedded (FFPE) slides were deparaffinized and rehydrated using xylene, followed by absolute ethanol, and then washed in tap water; endogenous peroxidase activity was quenched incubating slides with methanol containing 10% H_2_O_2_ for 15 min. Antigen retrieval was performed by heating the slides in a steamer for 20 min in Retrieve All 2 buffer (pH 10.0) (Covance, Princeton, NJ, USA). Then, incubation with UltraVision protein block (Thermo Fisher Scientific, Waltham, MA, USA) for 8 min was made to block nonspecific staining. The sections were then incubated overnight at 4 °C with the mouse monoclonal primary antibody vs. SARS-CoV-2 viral nucleocapsid protein (NC, 1:100, Sino Biological Inc., Beijing, China) diluted in PBS + 1% bovine serum albumin (BSA, Sigma-Aldrich, St. Louis, MO, USA). After rinsing with phosphate-buffered saline (PBS) 1M + 0.05% Tween20 (PBS-T; both Sigma-Aldrich), slides were incubated for 30 min at room temperature with goat vs. mouse horseradish peroxidase (HRP)-conjugated secondary antibody (EnVision, DAKO, Santa Clara, CA, USA). Specimens were developed using 3,3-diaminobenzidine tetrahydrochloride (DAB substrate kit, AbCAM, Cambridge, UK), counterstained with Gill’s hematoxylin N°2 (Sigma-Aldrich, St. Louis, MO, USA), and mounted using EuKitt (Bio-Optica, Milan, Italy). As negative controls for NP, slides from SARS-CoV-2 uninfected placentas (*n* = 2) were used. Pictures were captured using an Eclipse E800 microscope equipped with a cooled digital camera (DS-U1) and analyzed using LuciaG 5.0 software (all from Nikon Inc., Tokyo, Japan).

### 2.5. RNA In Situ Hybridization

Single-molecule RNA in situ hybridization SARS-CoV-2 RNA was detected by using RNAscope technology (Advanced Cell Diagnostics, Inc., Newark, CA, USA), an RNA in situ hybridization (ISH) technique. Paired double Z oligonucleotide probes were designed for hybridization to the target RNA by using custom software. The RNAscope 2.5 LS Probe V-nCoV2019-S (catalog number 848568; Advanced Cell Diagnostics) was used. The RNAscope 2.5 LSx Reagent Kit-Brown (Advanced Cell Diagnostics) in combination with a BOND-III Automated stainer (Leica Biosystems, Wetzlar, Germany) was used to process the samples according to the manufacturer’s instructions. The FFPE tissue section samples were prepared according to the manufacturer’s recommendations. The RNA integrity of each sample was evaluated with a probe designed for hybridization specifically to the ubiquitin C and cyclophilin B housekeeping genes. The negative control background staining was evaluated using a probe specific to the bacterial *dapB* gene. Each punctate dot signal representing a single target RNA molecule could be detected with standard light microscopic analysis.

### 2.6. Double Staining Immunohistochemistry for CD163/RNA In Situ Hybridization for SARS-CoV-2

Immunohistochemical staining with NovocastraTM Liquid Mouse Monoclonal Antibody CD163 (10D6 clone) (Leica Biosystems, Buffalo Grove, IL, USA) was used to identify Hofbauer cells in the placenta, combined with RNAscope 2.5 LS Probe V-nCoV2019-S. The double staining was performed on BOND-III Automated stainer (Leica Biosystems) according to the manufacturer’s instructions.

### 2.7. RNAscope Image Acquisition and Data Analysis

Images were captured using Axio Zeiss Scope A1 microscope. RNA marker was analyzed based on the average RNA dot number per cell. RNA quantity was scored based on manual counting following RNAscope Reference Guide described as follows.

Staining results were categorized into five grades according to the number of dots visualized under the brightfield microscope: 0—no staining or less than 1 dot to every 10 cells (X40 magnification); 1+—1–3 dots/cell (visible at X20–X40 magnification); 2+—4–10 dots/cell, very few dot cluster (visible at X20–X40 magnification); 3+—>10 dots/cell; and more than 10% positive cells have dot clusters (visible at X20 magnification); and 4+—>10 dots/cell, and more than 10% positive cells have dot clusters (visible at X20 magnification).

## 3. Results

**Figure 1:**
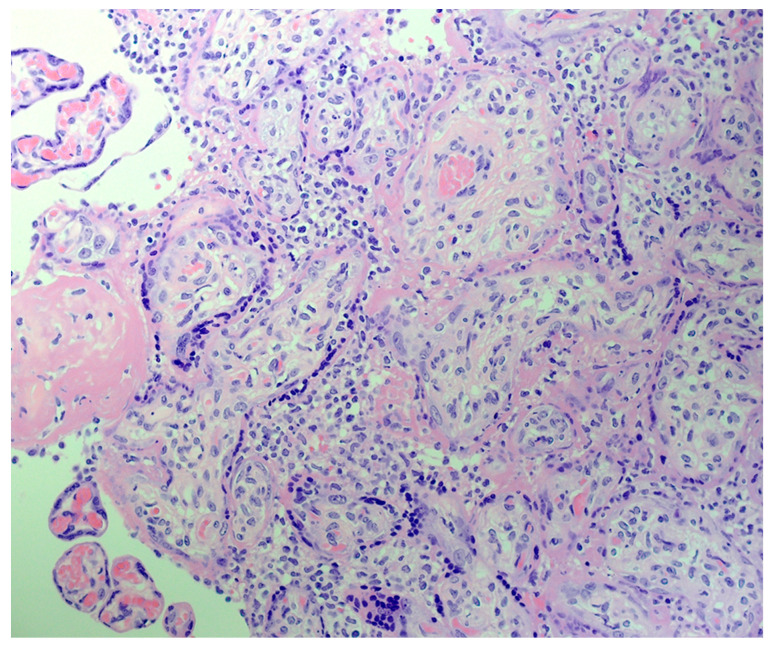
Placenta demonstrating chronic histiocytic intervillositis consisting of patchy histiocytic intervillositis with an accumulation of mononuclear inflammatory cells in the intervillous space. H&E. X40.

**Figure 2:**
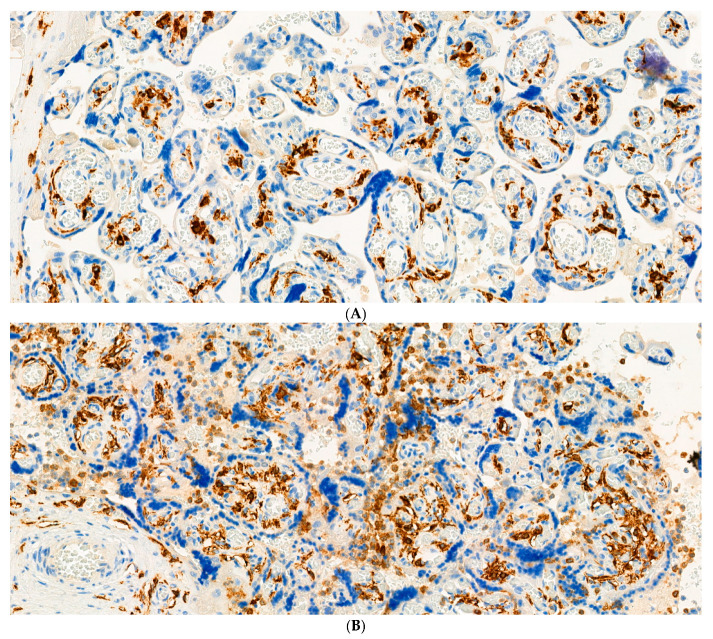
(**A**) In an area of non-inflamed placental tissue, the Hofbauer cells are seen as dark brown-staining, irregularly shaped, rounded, ovoid, and spindle-shaped cells in the chronic villous stroma using immunohistochemistry with antibody to CD163. X20. (**B**) An inflamed placenta, showing an increased number of Hofbauer cells in the villous stroma. There is chronic histiocytic intervillositis present in which the histiocytes in the intervillous space stain positively with this antibody. Antibody to CD163. X20.

**Figure 3:**
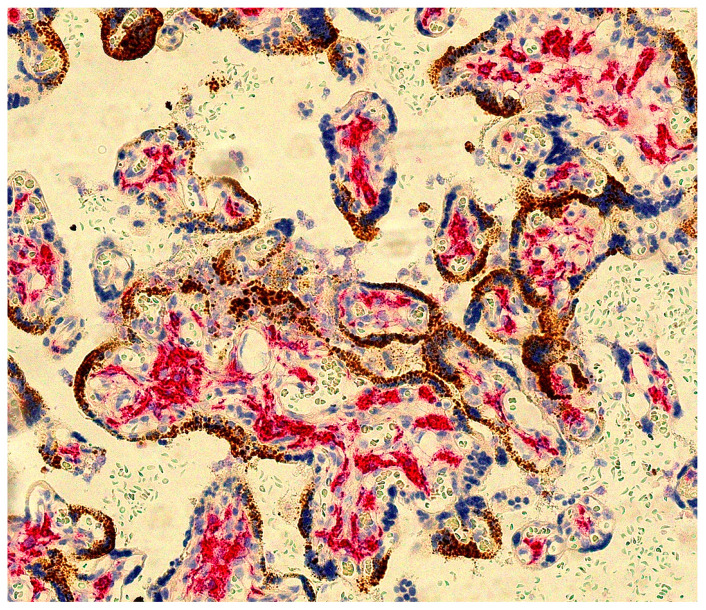
Hofbauer cells (red) are present in the stroma of these chorionic villi, but SARS-CoV-2 staining (brown) is restricted to the overlying syncytiotrophoblast. The Hofbauer cells appear to be increased in number in these villi, termed Hofbauer cell hyperplasia. Double staining with antibody to CD163 and SARS-CoV-2 RNAscope. X20.

The H&E-stained placental slides demonstrated chronic histiocytic intervillositis, characterized by collections of histiocytes within the intervillous spaces (Figure 1). These histiocytes in the intervillous space stained strongly positive using immunohistochemistry with anti-CD163. In the areas of intervillous histiocytic inflammation, the chorionic villi showed degeneration and necrosis of the syncytiotrophoblast. Immunohistochemical staining with antibody to CD163 also revealed the Hofbauer cells present in the chronic villous stroma (Figure 2). Hofbauer cells appeared as ovoid-to-spindle shaped cells with abundant cytoplasm staining positive for CD163 within varying locations in the chorionic villous stroma. Occasional Hofbauer cells were present in close proximity to the trophoblast basement membrane zone (Figure 2). In some areas, Hofbauer cells were a prominent constituent of the villous stroma, and in some villi, Hofbauer cell hyperplasia appeared to be present (Figure 2 and Figure 3).

**Figure 4:**
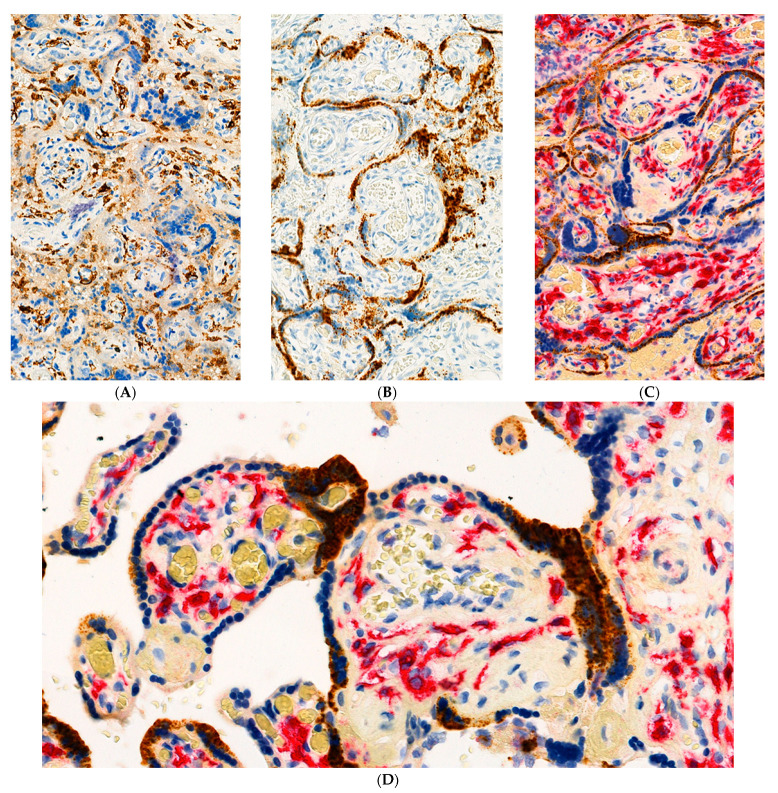
(**A**) Anti-CD163 clearly demonstrates the Hofbauer cells in chronic villous stroma and histiocytes in the intervillous spaces. Antibody to CD163. X20. (**B**) RNAscope in situ hybridization (ISH) staining is positive (brown dots) for SARS-CoV-2 spike protein mRNA in the villous syncytiotrophoblast. X20. (**C**) Double staining with antibody to CD163 (red) and SARS-CoV-2 ISH (brown dots). There is intense circumferential positivity of the syncytiotrophoblast for SARS-CoV-2 (granular brown staining) and absence of virus in the red staining Hofbauer cells. X20. (**D**) Double staining with antibody to CD163 (red) and SARS-CoV-2 ISH (brown dots). The absence of staining of Hofbauer cells for SARS-CoV-2 is clearly seen. Note the close proximity of some Hofbauer cells to the overlying infected trophoblast layer. X40.

**Figure 5:**
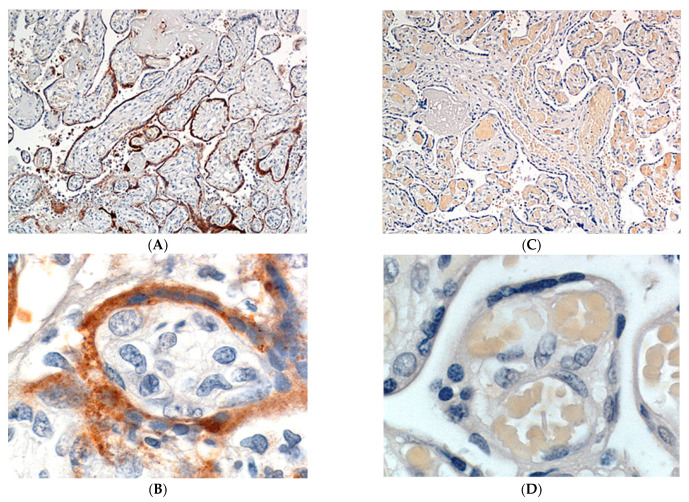
(**A**,**B**) Immunohistochemical expression of SARS-CoV-2 nucleocapsid (NC) protein in chorionic villous syncytiotrophoblast. The NC protein staining is present in the syncytiotrophoblast later of the chorionic villi, resulting in a pattern of circumferential villous staining. Antibody to SARS-CoV-2 nucleocapsid protein. (**A**), X10, (**B**), X100. (**C**,**D**) Placenta from a mother and fetus that were uninfected with SARS-CoV-2. Immunostaining using an antibody to SARS-CoV-2 nucleocapsid protein is completely absent. Antibody to SARS-CoV-2 nucleocapsid protein. (**C**), X10. (**D**), X100.

Sections were evaluated using RNAscope technology with the V-nCoV2019-S probe for SARS-CoV-2 spike protein mRNA. This revealed strong positivity for SARS-CoV-2 nucleic acid in villous syncytiotrophoblast (Figure 3 and Figure 4) of up to Grade 4+ according to the grading classification scale that was previously described. Immunohistochemistry using mouse monoclonal primary antibody against SARS-CoV-2 viral nucleocapsid protein also revealed strong positive staining of the syncytiotrophoblast (Figure 5). In those chorionic villi demonstrating infection, the staining for SARS-CoV-2 was present partially or completely circumferentially around the chorionic villi. The intensity of positive staining using these methods was suggestive of a large intracytoplasmic viral load within the infected syncytiotrophoblast cells (Figure 3 and Figure 4). Samples from uninfected control placentas did not show reactivity for SARS-CoV-2 (Figure 5C,D).

Meticulous analysis of the Hofbauer cells at moderate and high magnifications failed to demonstrate any evidence of staining for SARS-CoV-2 using the RNAscope probe. In some microscopic fields, Hofbauer cells closely approached the basement membrane zone of the infected trophoblast, but there was no staining for SARS-CoV-2 present in the macrophages (Figure 3 and Figure 4).

In addition to positive staining for SARS-CoV-2 in syncytiotrophoblast, there were also rare inflammatory cells in the maternal intervillous space that stained positively for SARS-CoV-2.

Except for chronic histiocytic intervillositis, there were no other inflammatory abnormalities present in this placenta. There was no chorioamnionitis, funisitis, or villitis present.

## 4. Discussion

Chorionic villus stromal macrophages, termed Hofbauer cells, are an important component of the immune system of the placenta. Hofbauer cells were first described in the 1980s by Castelucci et al. [21,22] and are located in the chorionic villous stroma, adjacent to both the fetal villous capillaries and the overlying trophoblast layer [21]. It is believed that these placental macrophages serve multiple purposes—host defense, villous development, vasculogenesis, and stromal maturation, and using their production of VEGF and FGF2 to have a role in clearing apoptotic cells. These immunologically active macrophages exhibit immunophenotypic and functional polarization and diversity that can vary with the gestational period. The majority of studies indicate that the epigenetic, antigenic, and functional features of Hofbauer cells most closely resemble alternatively activated macrophages that have been termed M2a, M2b, M2c, and M2d polarity subtypes [22,23]. Generally believed to be of fetal origin, Hofbauer cells are more abundant during the 1st and early 2nd trimester of pregnancy than in later gestation. The physical location of Hofbauer cells in close proximity to chorionic villus vessels, as well as the trophoblast layer, is significant in cases of potential transfer of substances and microbial agents across the maternal-placental interface [20,24,25,26].

When the placenta becomes infected or develops inflammation, Hofbauer cells may respond by producing pro-inflammatory cytokines or mediators. This reaction can result in damage to the villous maternal-placental barrier, and cause a villous fibrotic response associated with chronic inflammation [22]. There is no evidence that Hofbauer cells can become classically activated or develop an M1 polarity phenotype that can kill microbes [22,23,27], and some authors believe that the persistent M2-like properties of Hofbauer cells may be responsible for the observation that these cells appear to be ineffective in controlling most TORCH infections [22].

Hofbauer cells are implicated in a number of infectious diseases that affect pregnant women, and have been identified as targets for some viruses, protozoans, and other pathogens [20,28,29]. Hofbauer cells have also been found to be increased in number, and in some cases proliferate, in cases of intrauterine placental infection caused by a variety of TORCH agents, as well in placentas having villitis, an inflammatory abnormality of the chorionic villi having a number of different etiologies [24,25,28,29,30,31,32,33]. In particular, Hofbauer cells appear to play a role in some viral infections that can be transmitted transplacentally from mother to fetus—these include HIV [34,35] and Zika virus [30,36]. During the recent Zika virus pandemic that resulted in intrauterine infections and fetal malformations, the virus was found to localize within Hofbauer cells in placentas from infected fetuses [29,30,36,37].

It has been established that SARS-CoV-2 can be transmitted from an infected pregnant woman through the placenta to her fetus [10,11,12,13,14,15,18,19], although cases of intrauterine transmission are believed to account for a minority of neonates testing positive for the virus [38,39,40]. Pathology findings appear to demonstrate marked differences between those placentas that are infected with SARS-CoV-2 and transmit the virus and uninfected placentas from non-transmitting maternal-fetal dyads [18]. The findings in placentas from SARS-CoV-2-infected maternal-fetal dyads are distinctive, and include the occurrence of an unusual inflammatory lesion, chronic histiocytic intervillositis, accompanied by syncytiotrophoblast necrosis and intense positivity of syncytiotrophoblast for SARS-CoV-2 [19]. The use of molecular diagnostic techniques in placental analysis, including immunohistochemistry and RNA in situ hybridization to identify and localize SARS-CoV-2 antigens and nucleic acid, respectively, has been especially important in understanding the pathology spectrum from placentas that transmit SARS-CoV-2 [17].

Because of the relationship between some viruses and Hofbauer cells in cases of placental infections, the authors believed this case of transplacental maternal-fetal infection with SARS-CoV-2 was an opportunity to examine whether Hofbauer cell infection was occurring, especially given the intensity of viral staining in the syncytiotrophoblast. A thorough analysis of placental tissues using double-staining for macrophages and SARS-CoV-2 antigens and nucleic acid failed to locate any Hofbauer cells with intracellular viral positivity. Interestingly, in some areas, the Hofbauer cells within the villous stroma were in very close physical proximity to the basement membrane zone of the overlying infected trophoblast layer, in some cases abutting it. However, no viral staining of either Hofbauer cells or any other villous stromal cells was present. Considering the diffuse and intense staining of the syncytiotrophoblast for SARS-CoV-2 protein and RNA, it would appear that—at least, in this case, Hofbauer cells may not be a primary cell type involved in the maternal-fetal transmission of SARS-CoV-2.

Among the published cases of placentas having SARS-CoV-2 present in the syncytiotrophoblast using immunohistochemistry and/or RNA in situ hybridization, Hofbauer cell positivity has not been observed with the exception of one placenta. In a recent analysis of placentas from 6 liveborn neonates having been infected via transplacental transmission of SARS-CoV-2, molecular pathology analysis demonstrated high levels of staining for syncytiotrophoblast in all six, but no positivity of Hofbauer cells was noted in 5 of them despite intrauterine fetal infection [19]. In a single placenta, there were rare villous stromal cells—Hofbauer cells—that stained positive using immunohistochemistry for SARS-CoV-2 nucleocapsid protein, but did not stain for the spike protein [12,19]. The significance of this interesting finding from this one placenta is—it may represent productive viral infection occurring within the Hofbauer cells, or it could result from scavenged phagocytosed viral products. This same investigation [19] also examined the placentas from 5 stillborn or aborted fetuses with placental infection from SARS-CoV-2, all of which had syncytiotrophoblast infection with SARS-CoV-2, but no infection observed within Hofbauer cells. In addition to these cases of intrauterine transmission and fetal infection, a report of an uninfected neonate delivered to an asymptomatic mother with SARS-CoV-2 infection demonstrated chronic histiocytic intervillositis, syncytiotrophoblast necrosis, the positivity of the syncytiotrophoblast for SARS-CoV-2 by both immunohistochemistry and RNA in situ hybridization, but no staining of villous stromal cells or Hofbauer cells [41]. Based upon these findings from a limited number of cases, it appears that although SARS-CoV-2 may be present in Hofbauer cells in a small number of infected placentas, transplacental transmission of the virus and fetal infection may occur even in the absence of Hofbauer involvement, as occurred in our report. The relationship between Hofbauer cells and SARS-CoV-2 infection is clearly a topic that requires further investigation.

Cells that are infected with SARS-CoV-2 have in common the expression of angiotensin-converting enzyme 2 (ACE2). ACE2 is a transmembrane protease that responsible for the conversion of Angiotensin 1–8 (Ang II) to Angiotensin 1–7 (Ang 1–7) and is abundantly expressed in a wide range of cells from many different human organs, including some cells composing the maternal-fetal interface [42]. ACE2 is especially significant as it is the binding receptor of SARS-CoV-2 viral spike glycoprotein. Macrophages constitute a heterogeneous population of cells, and although the ACE2 receptor has not been identified in some tissue-residing macrophages [43], it has been found in others in such sites as the lung, spleen, and lymph nodes [44,45,46]. The ACE2 receptor for SARS-CoV-2 has not been identified to occur in Hofbauer cells in two studies using immunochemistry, although it is present in syncytiotrophoblast and cytotrophoblast cells [47,48]. Transmembrane serine protease 2 (TMPRSS2), a protease that cleaves the SARS-CoV-2 spike protein to facilitate infection and is necessary for viral binding to ACE2, membrane fusion, and cell entry, is also not present on Hofbauer cells [47].

There have been tremendous advances made in the understanding of the placental pathology of SARS-CoV-2 infection, the spectrum of pathological findings and the identification of some as risk factors for placental and fetal infection, and the potential role of the placenta in maternal-fetal infection [49]. It is important to determine what function, if any, that Hofbauer cells may have in both placental infections, as well as facilitating or inhibiting maternal-fetal transplacental transmission with SARS-CoV-2. Such studies are challenging to perform because the number of infected placentas and cases of transplacental maternal-fetal transmission of SARS-CoV-2 available for investigation remains limited. Our study, which is the first that was designed specifically to investigate the presence of SARS-CoV-2 in Hofbauer cells using molecular pathology methods, together with other cases that have been reported, suggests that maternal-fetal transmission of the virus can occur in the absence of Hofbauer cell involvement. Unlike some other infections that are transmissible through the placenta in which the Hofbauer cells are involved with the intrauterine transmission, preliminary evidence suggests that although SARS-CoV-2 may be present within Hofbauer cells, this finding is not necessary for transplacental transmission of the virus to occur.

## Data Availability

Data sharing is not applicable to this article.
